# The blood parameters and liver function changed inconsistently among children between burns and traumatic injuries

**DOI:** 10.7717/peerj.6415

**Published:** 2019-02-11

**Authors:** Chan Nie, Tao Wang, Huiting Yu, Xue Wang, Xueqin Zeng, Zairong Wei, Xiuquan Shi

**Affiliations:** 1 Department of Epidemiology and Health Statistics, School of Public Health, Zunyi Medical University, Zunyi, Guizhou, China; 2 Burns & Plastic Surgery, Affiliated Hospital of Zunyi Medical University, Zunyi Medical University, Zunyi, Guizhou, China; 3 Center for Injury Research and Policy & Center for Pediatric Trauma Research, The Research Institute at Nationwide Children’s Hospital, The Ohio State University College of Medicine, Columbus, OH, United States of America

**Keywords:** Burn, Children, Traumatic injury, Liver enzyme, Blood routine

## Abstract

**Objective:**

Burn and traumatic injury are two kinds of injury by modality. They cause acute phase response and lead to a series of pathological and physiological changes. In this study, we explored whether there are differences in routine blood parameters and liver enzyme levels between burned and traumatically injured children.

**Methods:**

Patients under 18 years old with injuries were recruited. Their demographic and clinical data were recorded. Collected clinical data included routine blood parameters (white blood cell count (WBC), red blood cell count (RBC), platelets (PLT), hemoglobin (HB)), serological enzyme levels (alanine aminotransferase (ALT), aspartate transaminase (AST), glutamyltransferase (GGT), alkaline phosphatase (ALP), cholinesterase (CHE)), and total protein (TP) levels (albumin (ALB), globulin (GLB)). A generalized linear model and multivariate analysis of variance were used to conduct comparisons.

**Results:**

A total of 162 children (109 with burns and 53 with traumatic injuries) with a mean age of 4.36 ± 4.29 years were enrolled in the study. Burned children had higher levels of RBC, HB, WBC, AST and lower levels of TP, CHE, ALB than traumatically injured children (*P* < 0.05). Moreover, the concentration of WBC and HB was higher in males compared to females (*P* < 0.001). Conversely, the level of AST and TP in males was lower, AST levels were significantly lower in males (*P* = 0.005). Age positively correlated with the levels of HB, AST and TP (*P* < 0.001), and negatively correlated with WBC (*P* < 0.001). With decreasing body mass index (BMI), the levels of WBC, HB, AST and TP significantly increased in both groups of injured children (*P* < 0.001). In addition, ISS was positively correlated with WBC and HB levels (*P* < 0.001), but negatively correlated with AST and TP levels (*P* < 0.001).

**Conclusions:**

Children with burn injuries suffered a greater acute response and liver damage than traumatically injured children. This may in part underlie clinical observations of differences in children morbidity and mortality in response to different injury types.

## Introduction

Epidemiologic analysis has long identified that children have a high risk of injury. Burns are the third to fifth leading cause of unintentional injury in children, and traumatic injury is the second most common cause of unintentional injury (Nie et al., 2017; Ogilvie et al., 2014; Shi et al., 2016; Shi et al., 2014; Sminkey, 2009). Both injury types are sudden attacks by external forces that damage the body. Burns refer to skin or other tissue damage caused by flames, hot liquid, electricity, or chemicals. Traumatic injury is a body wound or shock produced by sudden physical collision or movement. Once injured, the body undergoes an acute response, which can encompass a wide range of endocrinological, immunological and haematological effects (Krepska, Hastings & Roodenburg, 2017).

The acute phase response (APR) is a complex systemic early-defense mechanism activated by trauma, thermal injury, infection, stress, or drug exposure (Ceron, Eckersall & Martýnezsubiela, 2010). APR local reactions, initiated via neural signals, create proinflammatory cytokine (IL-1, IL6, TNF-α, and Neutrophil activation), and activate liver hepatocytes, modulating protein synthesis. APR induces leukocytosis, complement activation, protease inhibition and clotting, which serve to prevent infection, clear potential pathogens, initiate inflammatory processes, and contribute to the healing process (Borek, 1987; Carolyn, Julia & Altman, 2009; Ceron, Eckersall & Martýnezsubiela, 2010). During APR, immune system components are redistributed in order to adapt to the defensive state of the body. Studies have shown that during APR, the body’s defense system quickly mobilizes, exciting the sympathetic-adrenal medulla system. By mobilizing the action of catecholamines, glucocorticoids, and cytokines, blood cells in bone marrow reservoirs or blood edge pools mobilize to enter blood circulation, thereby increasing peripheral RBC, WBC and PLT counts (Chiu, Carlson & Lilly, 2007; Korkmaz et al., 2017; Lord et al., 2014; Pepe & Barba, 1999). As the detection of changes in blood biochemical indicators is relatively simple in clinical practice, and quantitative values can be obtained directly to reflect the physiological changes of injured patients. According to the change in blood biochemical indices, doctors can judge the injury characteristics of patients, give symptomatic treatment, and achieve the goal of wound healing. Thus blood biochemical indices of injured patients are of great significance.

In this study, we aim to test whether there are differences in blood parameters and liver enzyme levels between burned and traumatically injured children.

## Participants and Methods

### Participants

A cross-sectional comparative study was conducted at the Affiliated Hospital of Zunyi Medical University from June 1st, 2016 to May 31st, 2017. Patients under 18 years old, admitted to the hospital within 24 h of injury (including burns and other traumatic injuries), and who had completed a routine blood and serological enzyme examination within 1 day of admittance were eligible for enrollment in the study. Excluding criteria included the time interval between injury and hospital admission exceeding 24 h, a history of other diseases before admission, prior other hospital visits, prior therapy (except for cleaning and dressing wounds), and continued treatment of a previous injury (such as scar treatment after burn healing or hospitalization after hospital discharge). Patients from the burn center, surgery departments, and even the ICU (Intensive Care Unit) were enrolled in the study.

### Data collection

Clinical data were obtained directly from each department before patients were discharged from the hospital, including routine blood parameters (white blood cell count (WBC), red blood cell count (RBC), platelets (PLT), hemoglobin (HB)), serological enzyme levels (alanine aminotransferase (ALT), aspartate transaminase (AST), glutamyltransferase (GGT), alkaline phosphatase (ALP), cholinesterase (CHE)), and total protein (TP) levels (albumin (ALB), globulin (GLB)). Injury severity score (ISS) was calculated by adding the squares of the three highest abbreviated injury scale (AIS) grades in each of the three most severely injured body regions, and values range from 1 to 75 (Citerio, 2012; Copes et al., 1988). ISS was determined by the lead doctor in our study. Percent total body surface area (TBSA) burned, demographic data including age, gender, height, weight, and other basic information about the injury including time, location, and cause of injury were collected by in-person surveys. Some missing data were obtained by a follow-up call.

### Statistical analysis

Epidata 3.0 was used to build the database. Data was analyzed with SPSS 18.0 (SPSS Inc., Chicago, IL, USA). Univariate analysis of variance was used to determine the difference of blood components in injury and gender groups. Age was adjusted (as a covariate in the Univariate analysis) in this study, since children of different ages generally have different blood parameters. Multivariate analysis of variance was used to analyze liver enzymes in burn and traumatic injury groups, as these enzymes are related to each other and are useful for the clinical diagnosis of liver dysfunction. A generalized linear model was used to examine the possible factors affecting WBC, HB, AST and TP. A *p*-value of <0.05 was considered statistically significant.

## Results

### Characteristics of children with burns and traumatic injuries

During the study period, 162 hospitalized children with a mean age of 4.36 ± 4.29 years were enrolled and included in the analysis. Of the 162 children, 109 suffered burns and 53 suffered traumatic injuries. Both burn and traumatic injury groups were dominated by males. The ratio of male to female children with burns and with traumatic injuries was 69:40 and 35:18 respectively. The etiology of burns in children was mainly hot liquid (76.1%), followed by fire-related factors (14.7%). In the traumatic injury group, children mostly suffered from a fall (67.9%), followed by traffic injuries (13.2%). Limb injuries occurred most frequently in children from both groups. ISS scores ranged from 1 to 25 (3.68 ± 4.36) in the burn injury group, and 4 to 21 (6.76 ± 3.51) in the traumatic injury group. Most of the injuries were moderate, so the ISS scores were not high.

### Blood routine in burned and traumatic injury children

Blood test was conducted within 1 day of patient’s admission to the hospital. According to univariate analysis of variance, the mean HB and WBC concentration in burned children was significantly higher than that in traumatically injured children (*P* < 0.05), regardless of age adjustment. The mean PLT concentration in burned children was significantly higher than that in traumatically injured children (*P* = 0.001), however after age adjustment it was not significant (*P* = 0.074) (Table 1). In contrast, the mean RBC concentration in burned children was statistically significantly higher than that of traumatically injured children (*P* < 0.001) only after age adjustment. Only the mean TP concentration was lower in burned children than in traumatically injured children regardless of age adjustment (*P* < 0.001) (Table 1). Blood components were not significantly different between genders (Table 1).

**Table 1:** Blood parameter routine examination in different injury groups.

Indices^a^	HB	PLT	WBC	RBC	TP
Burn (*n* = 109)	125.12 ± 16.10	372.14 ± 126.80	16.31 ± 7.41	4.80 ± 0.56	58.99 ± 7.94
Non-burn (*n* = 53)	117.91 ± 13.93	304.85 ± 88.84	11.02 ± 3.95	4.20 ± 0.51	66.31 ± 7.92^b^
*P*-value	0.005	0.001	<0.001	0.25	<0.001
Age-adjusted *F*-value	15.785	3.241	15.245	30.507	21.285
Age-adjusted *P*-value	<0.001	0.074	<0.001	<0.001	<0.001

**Notes.**

Differences in HB, WBC, RBC and TP between burn and non-burn group were statistically significant when age adjusted.

a
 HB hemoglobin (g/L) PLT platelet (10^9^/L) WBC white blood cell (10^9^/L) RBC red blood cell (10^12^/L) TP total protein (g/L)

Values expressed as mean ± SD.

b *n* = 47.

### Liver function (enzyme and total protein) examination in burned and traumatically injured children

According to multivariate analysis of variance, liver enzymes and total protein concentrations were significantly different between the two injury groups (*P* < 0.05) (Table 2). Of the five enzymes, AST concentration was significantly higher in burned children (*P* < 0.05), ALT was slightly higher in the burn injury group (*P* > 0.05); GGT, ALP and CHE were higher in traumatically injured children than burned children, CHE with significant difference (*P* < 0.05) (Table 2). Both ALB and GLB total protein concentrations were higher in traumatically injured children, ALB with significant difference (*P*  <  0.05) (Table 2).

**Table 2:** Liver enzyme (U/L) and total protein (g/L) in different injury groups.

Groups	*n*	Liver enzyme					Total protein	
		ALT	AST	GGT	ALP	CHE	ALB	GLB
Burn	109	17.73 ± 10.92	42.84 ± 27.54	11.21 ± 5.06	235.55 ± 96.19	7.52 ± 2.11	38.15 ± 5.81	20.84 ± 4.50
Non-burn	47	16.38 ± 11.16	33.89 ± 9.71^a^	12.23 ± 3.73	255.04 ± 89.15^b^	8.14 ± 2.44^a^	42.86 ± 5.52^a^	23.45 ± 3.93
*F*-value	3.663						14.141	
*P*-value	0.004						<0.001	

**Notes.**

ALT alanine aminotransferase AST aspartate transaminase GGT glutamyltransferase ALP alkaline phosphatase CHE cholinesterase ALB albumin GLB globulin

Compared to the burn group, ^a^ *P* <  0.05, ^b^*P* = 0.05.

### Factors affecting WBC, HB, AST and TP in injured children analyzed by a generalized linear model

Blood indices with significant differences between burn and traumatic injury groups were analyzed by a generalized linear model. Factors possibly affecting blood indicators were entered into the model, including gender, age, BMI (BMI = weight in kg/ (height in m)^2^), ISS and injury group. The concentration of WBC and HB in males was higher than females (*P* < 0.001); conversely, the level of AST and TP in males was lower, AST with significant difference (*P* = 0.005). Age was positively correlated with the levels of HB, AST and TP (*P* < 0.001), and negatively correlated with WBC (*P* < 0.001). With decreasing BMI, the level of WBC, HB, AST and TP significantly increased in both groups of injured children (*P* < 0.001). In comparison to traumatically injured children, burned children had higher levels of WBC, HB and AST (*P* < 0.001), but lower levels of TP (*P* < 0.001). In addition, ISS was positively related to WBC and HB (*P* < 0.001), but negatively related to AST and TP (*P* < 0.001) (Tables 3–4). It is worth noting that injury group was the most influencing factor for WBC, HB, AST and TP levels. Age and gender were the 2nd and 3rd most influencing factors for HB and AST, gender and ISS were the 2nd and 3rd most influencing factors for WBC, while ISS and BMI were the 2nd and 3rd most influencing factors for TP. Regarding the etiology of WBC in burned children, we found that children burned by hot oil bear the highest levels of WBC (*B* = 6.459, *P* < 0.001), compared to hot food (*B* = 4.043, *P* < 0.001), hot water and soup (*B* = 1.760, *P* = 0.001) or fire (*B* =  − 1.566, *P* = 0.002).

**Table 3:** Factors affecting WBC and HB among injured children.

Factors^a^	Indices	B	95% CI	Wald *χ*^2^	*P*-value
Male vs Female	WBC	0.700	0.375–1.024	17.868	<0.001
	HB	0.896	0.572–1.220	29.320	<0.001
Age	WBC	−0.305	−0.344–0.265	231.287	<0.001
	HB	1.004	0.965–1.043	2,514.409	<0.001
BMI	WBC	−0.166	−0.214–0.118	45.883	<0.001
	HB	−0.175	−0.223–0.127	51.347	<0.001
Burn vs Traumatic injury	WBC	6.249	5.884–6.614	1,126.205	<0.001
	HB	11.503	11.138–11.868	3,815.572	<0.001
ISS	WBC	0.666	0.627–0.705	1,115.439	<0.001
	HB	0.279	0.240–0.318	195.373	<0.001

**Notes.**

BMI Body Mass Index ISS Injury Severity Score

a The latter of two factors is the reference factor. B, regression coefficients; 95% CI, 95% confidence interval.

**Table 4:** Factors affecting AST and TP among injured children.

Factors^a^	Indices	B	95% CI	Wald *χ*^2^	*P*-value
Male vs Female	AST	−0.474	−0.808–0.140	7.749	0.005
	TP	−0.208	−0.542–0.126	1.494	0.222
Age	AST	1.058	1.017–1.098	2,642.405	<0.001
	TP	0.267	0.2270–0.307	168.440	<0.001
BMI	AST	−0.376	−0.424–0.328	236.472	<0.001
	TP	−0.306	−0.3540–0.258	156.962	<0.001
Burn vs Traumatic injury	AST	11.473	11.084–11.861	3,352.590	<0.001
	TP	−8.988	−9.3760–8.599	2,057.467	<0.001
ISS	AST	−0.457	−0.496–0.418	530.986	<0.001
	TP	−0.836	−0.8750–0.797	1,778.010	<0.001

**Notes.**

BMI Body Mass Index ISS Injury Severity Score

a The latter of two factors is the reference factor. B, regression coefficients; 95% CI, 95% confidence interval.

## Discussion

Blood routine analyses showed that the levels of HB and RBC were higher in burned children than traumatically injured children, while TP and ALB levels were lower. In the early stage of burn wounds, the permeability of microvessels can be significantly increased, resulting in massive fluids and protein extravasation, which causes a decrease in blood volume (Arlati et al., 2007; Haberal, Abali & Karakayali, 2010), thus the concentration of RBC and HB increases. Moreover, the super metabolism decomposition and weakening synthesis of protein in wounds leads to a further decrease in permeation pressure and total blood volume. It causes the protein in plasma to decrease, resulting in a significant reduction in the levels of ALB and TP, which in turn can cause the occurrence of hypoproteinemia (Cochran et al., 2007). In addition, most of the enrolled children were at a young age in our study. Their glomerular and renal tubular functions have not been well developed yet, thus their ability to maintain body fluid balances is poor, so they are more likely to lose fluid in certain conditions. It is worth noting that in our study, the mean age of the burn group is lower than the traumatic injury group (3.23 vs. 6.60). Although we have taken age as an adjusted factor in our analysis, our results would benefit from further evaluation.

Additionally, the level of WBC was higher in burned children. Leukocytes are the body’s main line of defense to defend against pathogens and other invading foreign bodies (Chertov et al., 2010; Friedl & Weigelin, 2008), thus an increased number of WBC is a typical response to acute inflammatory/traumatic stress. It seems that both burns and traumatic injuries can induce APR, however the acute response to burns is markedly greater. It has been observed that burn patients have a greater and more sustained immune-inflammatory response than traumatically injured patients (Mace et al., 2012; Valvis et al., 2015). This study’s results support the above observation, showing that WBC levels increased more in burned children.

It is well known that the serum enzymes ALT, AST, ALP and GGT contribute to the function of liver cells. When the hepatocyte membrane is damaged or the cells undergo necrosis, these enzymes enter the serum and as a result their concentration increases (Pimentel et al., 2018; Puranik et al., 2002; Solovyeva et al., 2013). In contrast, serum CHE is synthesized by the liver, thus a decrease in this enzyme often reflects liver damage (Ogunkeye & Roluga, 2006). The multivariate analysis of variance of our results showed liver enzymes were significantly different in burn and traumatic injury groups. AST was significantly higher in burned children and CHE was lower, which suggests that the liver damages were often concomitant in burned children. This might relate to the decreased hemagglutination in the early stage of burn wounds, causing hepatocyte ischemia and hypoxia.

The generalized linear model showed that the level of WBC and HB is higher in boys than girls, which is consistent with the fact that male blood cell counts are generally higher than female. In contrast, this study showed levels of AST in males to be lower than females, which might indicate that injury has a greater effect on liver damage in males than in females. We found age to be positively correlated with the concentrations of HB, AST and TP, but negatively correlated with WBC. Older children, with a more complete kidney function to maintain fluid and protein balances, had lower WBC and higher HB levels, however the influence of age on AST is not yet clear.

We suggest the negative correlation between BMI and WBC, HB, AST and TP is due to children with a lower BMI inducing a particular APR to trauma. On the other hand, ISS is well established in trauma patients to measure the severity of an injury (Citerio, 2012; Copes et al., 1988). Results showed that ISS was positively correlated with WBC and HB, but negatively correlated with AST and TP. It is reasonable to suggest that a severe injury would induce an immune response and a fluid imbalance disorder, causing the levels of WBC, HB and TP to change. However, the negative correlation between ISS and AST levels indicates that a severe injury may not cause liver damage.

Interestingly, we found that children burned by hot oil had the highest levels of WBC, followed by hot food and hot water or soup. We suggest this is because oil is more likely to adhere to the skin and the residual heat can be transmitted deep into the tissue. However food contains a variety of spices and other substances, and it is difficult to explain their role in the etiology of WBC in burned children.

## Conclusion

It is noteworthy that in our study the ISS in the traumatic injury group was higher than in the burn injury group, but the blood parameters and liver enzyme levels indicated that there was a greater APR and more severe liver damage in burn patients. It has been found that ISS is an independent risk factor for mortality in patients with a combination of burns and traumatic injuries; however, ISS is limited to predict outcomes of burn patients. Despite the limitation of the ISS, we concluded that burns play a more important role in APR and liver damage than traumatic injury. Subsequent research could explore the relationship of blood parameters and liver enzymes to other injury data, such as duration of hospital stay, presence of multiple organ dysfunction syndrome, mortality, etc., and further clarify the prognosis of burned and traumatically injured patients.

### Declaration of patient consent

The authors certify that they have obtained all appropriate patient consent forms. In the form, the patients have given their consent for their images and other clinical information to be reported in the journal. The patients understand that their names and initials will not be published and due efforts will be made to conceal their identity, but anonymity cannot be guaranteed.

##  Supplemental Information

10.7717/peerj.6415/supp-1 Supplemental Information 1 Dataset The raw data of our manuscript.

## Additional Information and Declarations

### Data Availability

The following information was supplied regarding data availability: The raw data are available in the Supplemental File.
